# CT is the new standard for the diagnosis of coronary artery disease in daily practice

**DOI:** 10.1007/s12471-024-01907-2

**Published:** 2024-10-07

**Authors:** José P. S. Henriques, R. Nils Planken

**Affiliations:** 1 Department of Cardiology, Amsterdam University Medical Centre, Amsterdam Cardiovascular Sciences, University of Amsterdam, Amsterdam, The Netherlands; 2 Department of Radiology, Amsterdam University Medical Centre, University of Amsterdam, Amsterdam, The Netherlands

Computed tomography coronary angiography (CTCA) is increasingly integrated into current guidelines and clinical practice. In the Netherlands, the uptake of CTCA in clinical practise is low [1]. Currently, CTCA practice in the Netherlands is characterized by heterogeneous standard operation procedures and quality standards precluding optimal usage in the clinical field. National trend analyses in the Netherlands and the United Kingdom indicate a substantial increase in CTCA usage in the near future up to at least 300% [1]. We need to change our current practise for diagnosing coronary artery disease and change does not come easy. Therefore this special issue of the Netherlands Heart Journal focuses on the current evidence, standards and practices of CTCA, shedding light on ongoing studies as well as future and upcoming developments across six chapters. These papers cover topics such as the early detection of coronary artery disease [2], the implementation of the CAD-RADS reporting system [3], and its application in trials such as the CLEAR-CAD trial [4]. The CLEAR-CAD trial is the largest clinical trial in the Netherlands, currently investigating a combined CT-first diagnostic and optimal medical treatment strategy for the clinical assessment of chest pain in the outpatient setting.

Many chest pain patients in the Netherlands are still not undergoing the guideline-recommended CTCA. A significant proportion of patients in the outpatient setting undergo exercise ECG stress testing (48%), chest X‑rays (38%) or invasive coronary angiography (31%). Additionally, due to different diagnostic pathways, a substantial group of patients are being treated with optimal medical therapy after revascularisation or undergoing revascularisation (36–63%) [5].

There are, however, threats for the swift adoption of CTCA for outpatient chest pain such as a reduced capacity of CT scanners, radiologists and radiology technicians.

But there is more to this issue of CTCA. While we await the further adoption of the CAD-RADS system and the results from the CLEAR-CAD trial, we must expand our horizon to the future. What could be the role of CTCA for asymptomatic patients? Interestingly, despite coronary artery disease being the leading cause of death, no screening programs for its detection currently exist. This contrasts with screening programs for various malignancies. This will be particularly important in the coming years as the population ages. A first glimpse into this area can be expected from the SCOT-HEART 2 trial, which will recruit at least 6000 asymptomatic individuals from Scotland who are at high risk of heart disease. However, it will take many years before we can expect outcome data from this trial.

What about further developments, such as the addition of computed tomography-derived fractional flow reserve (CT-FFR) for lesion severity assessment [6], which is being studied in two ongoing randomised clinical trials in the Netherlands (the Fusion trial and the iCORONARY trial)? The deployment of CT-FFR may further reduce the need for functional testing, which remains an integrated part of guidelines and the CLEAR-CAD trial.V.

Moving from software to hardware, the clinical introduction of photon-counting CT opens up a radical new level of accuracy, potentially reducing the need for CT-FFR software or improving CT-FFR diagnostic accuracy [7]. This technology paves the way for assessing even complex coronary artery disease and applications, including post-PCI stent patency.

The highly accurate assessment of coronary artery disease does not end with photon-counting technology. In this era of artificial intelligence, a glimpse into the potential of using AI in this area is demonstrated [8]. Adding AI to current CTCA alone already offers opportunities for a fully automated (pre)assessment modality. These machine learning tools may quickly not only qualify and quantify coronary artery disease in great detail but also expand to a fully automated assessment of the entire scan range, including the complete evaluation of the acquired chest and upper abdomen images, thereby increasing individual risk assessment beyond coronary artery disease.

We are truly entering a new era where we transition from invasive coronary angiography (with or without invasive FFR) to a CTCA-based diagnosis of coronary artery disease (with or without CT-FFR) to rule out obstructive coronary artery disease. Additionally, CT will become an increasingly important modality for the early detection of coronary artery disease, utilising full CTCA, including perivascular fat analysis, or perhaps even just a calcium score for asymptomatic patients. The introduction of innovative techniques, such as photon-counting CT and AI tools, will further enhance the capabilities and potential of non-invasive imaging in this field. The combined use of advanced hardware and software with AI tools will enable a complete and extremely accurate assessment of the coronary tree, including all its plaques and other components.

It is crucial to understand that this field is rapidly changing for both cardiologists and radiologists, and close collaboration within and between hospitals is key to planning how to adopt and implement the latest CTCA technology in clinical practice to best serve our patients.

We hope that readers find this comprehensive overview of CTCA both interesting and valuable.
